# MCAK/Kif2C centromeric activity level tunes K-fiber stability

**DOI:** 10.1101/2025.02.16.638494

**Published:** 2025-02-17

**Authors:** Linda Wordeman, Mike Wagenbach, Juan Jesus Vicente

**Affiliations:** 1 Department of Neurobiology and Biophysics, University of Washington School of Medicine, Seattle, WA, USA.

## Abstract

MCAK/Kif2C is a microtubule-depolymerizing kinesin that is implicated in the correction of chromosome attachment errors. When this protein is eliminated from kinetochores, cells exhibit delayed congression and a modest increase in chromosome mis-segregation. Curiously, MCAK/Kif2C overexpression (OE) promotes these same defects. These mitotic delays are restricted to prometaphase and can be rescued by modulating MCAK/Kif2C activity solely at the centromere. Both excessive depletion and surplus levels of centromeric MCAK/Kif2C increased inter-kinetochore distances (IKDs) commensurate with an increase in acetylated tubulin in the spindle, a readout for k-fiber stability. Because both high and low levels of centromere-associated MCAK/Kif2C increased k-fiber stability, we conclude that this is the likely mechanism for the increased chromosome segregation errors observed in both these antagonistic conditions. Loss of centromeric MCAK/Kif2C delayed the conversion from lateral to end-on motility was delayed in MCAK/Kif2C-depleted cells. This likely represents the key activity that MCAK/Kif2C imparts to the centromere which, when present at consistently incorrect levels, slows k-fiber turnover and congression.

## Introduction

MCAK/Kif2C is the most well studied member of the kinesin-13 family of microtubule (MT) depolymerizing kinesins. A comprehensive review of the protein's enzymology, structure and clinicopathology is available (Kreis et al., 2024). Briefly, MCAK/Kif2C is thought to use its MT depolymerizing activity to detach aberrant MT connections with the kinetochore (KT) but it has never been determined, mechanistically, how these connections are recognized and severed by the enzyme. Thus, there is still much to be learned about this process.

MCAK/Kif2C is present in the cytoplasm, nucleus and at the centrosome and in cells that are cycling but appears to be lost once they differentiate terminally or become senescent (Ginkel & Wordeman, 2000; Gwon et al., 2012) unless artificially reintroduced or retained in tissue-specific stem cells. MCAK/Kif2C protein levels increase slowly up through G2, reaching peak levels at mitotic prophase and then returning to a minimal level in recently divided G1 cells (Schweiggert et al., 2021). During mitosis, MCAK/Kif2C associates with a number of different cellular structures including spindle poles, kinetochores, midbodies and MT plus-ends (Honnappa et al., 2009; Wordeman & Mitchison, 1995). Studies have pinpointed a requirement for MCAK/Kif2C activity during congression to achieve metaphase chromosome alignment (Illingworth et al., 2010; Kline-Smith et al., 2004; Ritter et al., 2015).

MT regulators like MCAK/Kif2C present a challenge to study because when their levels are altered, tubulin homeostasis is perturbed triggering compensatory changes in tubulin expression (Gasic et al., 2019). We endeavored to circumvent this by changing MCAK/Kif2C levels (whenever possible) on a timescale that would not be permissive for compensatory cellular responses. To accomplish this, we have engineered CRISPR cells to express GFP-FKBP-MCAK/Kif2C under endogenous promoters. The protein can be relocalized in minutes in the presence of rapamycin to any subcellular structure that is associated with exogenously added blue fluorescent protein (BFP) conjugated to the FKBP12-rapamycin binding (FRB) domain. By studying the enzyme at physiological levels in cells we have been able to add some refinements to our understanding of MCAK/Kif2C's localization and behavior that differ a little from the published literature.

For example, in contrast to previous studies including our own, MCAK/Kif2C is not found on MT plus-ends (+TIP) except during mitotic prometaphase. We do not see it on interphase MTs in cells expressing endogenous levels of MCAK/Kif2C (Wagenbach et al., 2023). However, the protein can be artificially driven onto MT plus ends at any time during the cell cycle. We have found the quantity of +TIP MCAK/Kif2C is proportional to the cytoplasmic expression level of MCAK/Kif2C. Thus any cells that have been transiently transfected with MCAK/Kif2C expressing constructs over and above endogenous protein level will appear to have +TIP-associated MTs MCAK/Kif2C when they normally would not. Another way to artificially drive MCAK/Kif2C onto MT plus-ends is by ectopic expression of EB3, a protein not normally expressed in our cell lines. Interestingly, EB1, which is naturally expressed in our cell lines, does not artificially recruit MCAK/Kif2C to MT plus-ends even when overexpressed (Wagenbach et al., 2023).

We confirm that loss of MCAK/Kif2C results in an increase in lagging chromosomes at anaphase (Kline-Smith et al., 2004; Maney et al., 1998). However, because lagging chromosomes arise stochastically by, potentially, a variety of different mechanisms, this readout serves as a rather low-resolution method for determination of the mechanistic function of this protein. The limitation of the lagging chromosome assay is underscored by our observation of an increase in lagging chromosomes during both loss and overexpression (OE) of MCAK (Wagenbach et al., 2020). One would think that these two antagonistic experimental treatments would surely lead to functionally distinct outcomes. The goal of this study is to mechanistically explain why loss and overexpression have the same superficial results.

To avoid as many compensatory cell responses as possible we have used the rapamycin-based "knock-sideways" approach to remove MCAK/Kif2C from mitotic structures within a time scale of minutes (Wordeman et al., 2016). In this way we have been able to pinpoint with greater precision the time that MCAK activity is required during cell division: prometaphase. This is a time at which most, if not all, kinetochores are occupied by MCAK/Kif2C.

We have used our cell system to compare the mitotic effects of both MCAK/Kif2C loss and OE. While MCAK/Kif2C loss has been extensively investigated, OE is also worth investigating because it appears to increase errors in a manner that appears similar to MCAK/Kif2C loss (Wagenbach et al., 2020). Furthermore, the mitotic effect of MCAK/Kif2C OE has not been studied despite the fact that the protein is overexpressed in many cancers (Rodrigues-Ferreira et al., 2023) and has the potential to increase chromosome instability to promote tumor evolution.

We have found that both loss and OE of MCAK/Kif2C delays chromosome congression to the metaphase plate to the same extent and concomitant with increased stability of kinetochore fiber (K-fiber) microtubules. We believe that this occurs because increased inter-kinetochore distances are promoted by both conditions. Increased IKD might place pulling forces on the kinetochore fiber MTs. Such pulling forces have been implicated in suppressing MT disassembly and turnover (Akiyoshi et al., 2010).

MCAK/Kif2C OE is also correlated with a measurable but very small reduction in the fluorescence intensity of Mad2 positive kinetochores per cell. This is interesting because we have previously recorded higher Mad2 levels at centromeres during MCAK/Kif2C loss (Domnitz et al., 2012). Another principal difference between the loss and OE phenotypes is that kinetochores without MCAK/Kif2C appear delayed in biorienting while kinetochores with an excess of MCAK/Kif2C are not. This may explain the differential Mad2 behavior. Regardless, both conditions go on to promote increased K-fiber stability and chromosome segregation errors by promoting excess sister-centromere tension and increased interkinetochore distance (IKDs).

## Results

**Figure 1.**
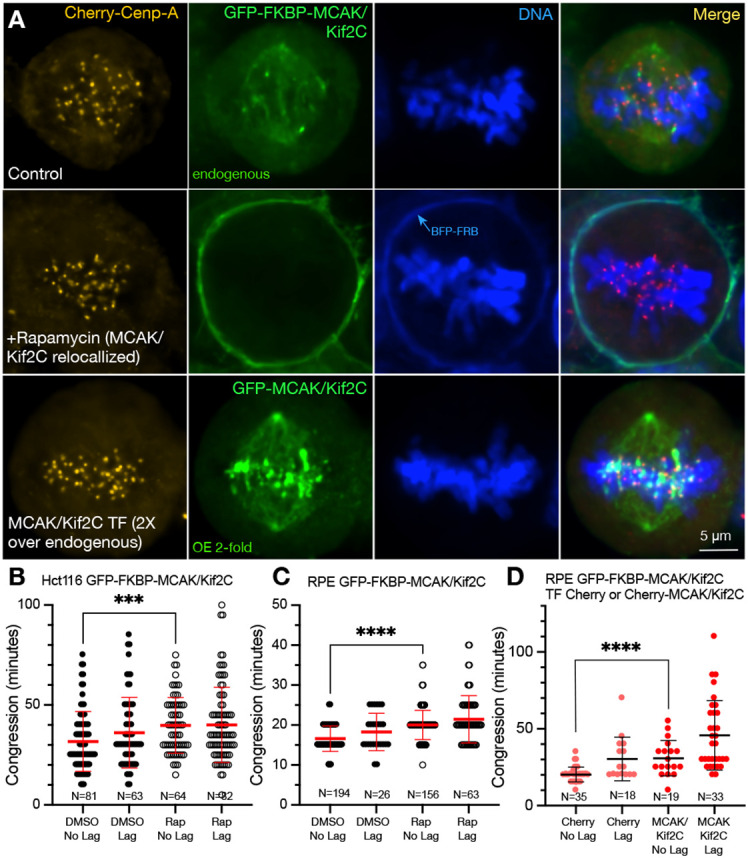
Live GFP-FKBP-MCAK/Kif2C cells exhibit delayed congression from NEB to metaphase when there is either too much or too little MCAK/Kif2C associated with the mitotic spindle. **A.** GFP-FKBP-MCAK/Kif2C parent line co-engineered with cherry-CENP-A (top). GFP-FKBP-MCAK/Kif2C parent line stably expressing BFP-FRB-SH in the presence of Rapamycin (middle) which relocalizes MCAK/Kif2C to the plasma membrane. An example of the GFP-FKBP-MCAK/Kif2C parent line transiently transfected with exogenous GFP-MCAK/Kif2C (bottom). The fluorescence level is 2-fold what would normally be seen in the parent cell line. **B.** Live Hct116 GFP-FKBP-MCAK/Kif2C cells followed through mitosis in the presence or absence of rapamycin. **C.** Live RPE GFP-FKBP-MCAK/Kif2C cells followed through mitosis in the presence or absence of rapamycin. **D.** Live RPE GFP-FKBP-MCAK/Kif2C transiently transfected with Cherry-MCAK/Kif2C. Cells in B,C and D were followed through mitosis using Hoechst 33342.

We have engineered a cell line expressing GFP-FKBP-MCAK/Kif2C with stably integrated BFP-FRB-SH in the plasma membrane (Fig. 1A, **top**). We have treated this cell line in two different ways. By adding 1μm rapamycin we can rapidly relocalize GFP-FKBP-MCAK/Kif2C to the plasma membrane (Fig. 1A, **middle**). Although we find complete removal to the membrane to occur within minutes, we apply rapamycin for **4 hours** prior to imaging for consistency between experiments. Sometimes, partial relocation occurs in certain cells that have partially or completely lost their FRB moiety. We do not include such cells in our analyses. Alternatively, we can transiently transfect a plasmid coding for either GFP-MCAK/Kif2C or cherry-MCAK/Kif2C into cells expressing endogenous GFP-FKBP-MCAK/Kif2C (Fig. 1A, **bottom**). When we use two GFP-linked constructs in this manner (usually to accommodate another fluorescent moiety such as our cherry-CENP-A expressing cells) we limit our analysis to cells with integrated fluorescence density that is twice that of an untransfected control. This prevents us from evaluating cells that are experiencing extreme excess of MCAK/Kif2C while still enabling the reliable identification of transfected cells. We track and stage cells throughout cell division using fluorescently labeled DNA or a fluorescently CRISPR-engineered centromere marker CENP-A.

Live tracking of mitotic cells shows that both excess MCAK/Kif2C and loss of MCAK/Kif2C delays the time required to progress from nuclear envelope breakdown (NEB) to the metaphase plate to a similar extent (Fig. 1). On average, delayed congression is a regular feature of cells that go on to exhibit lagging chromosomes at anaphase (Fig. 1B-D, **"Lag"**). However, we have found that congression is also delayed in cells that do not go on to exhibit lagging chromosomes but whose centromeres either lack MCAK/Kif2C (rapamycin-treated) or are associated with excess MCAK/Kif2C (transfected with either cherry-MCAK/Kif2C (Fig. 1D) or GFP-MCAK/Kif2C (Fig. 1A, **bottom**)). Statistical comparisons between parsed live cells that progress through cell division with no lagging chromosomes (Fig. 1B-D, **Mann-Whitney t-test, "No Lag"**) are shown.

Lagging chromosomes during anaphase are thought to primarily arise from merotelic attachments between one sister-kinetochore and MTs emanating from both centrosomes. These connections arise naturally during the course of chromosome attachment to the mitotic spindle and congression (Cimini et al., 2003). They are resolved prior to anaphase or even during anaphase in a mechanistically interesting but somewhat opaque manner (Cimini et al., 2004).

It is well-established from a number of studies that loss of MCAK/Kif2C can lead to a modest increase of lagging chromosomes during anaphase. This led to a conviction within the mitotic community that MCAK/Kif2C is required to detach MTs from merotelic connections. MCAK/Kif2C is a MT depolymerizer so this activity would be expected to participate in the detachment of excess/erroneous MT attachments. However, it is puzzling that when MCAK/Kif2C is only twice endogenous levels (as measured by fluorescence intensity), cells present the same defect: lagging chromosomes and delayed congression.

MCAK/Kif2C associates with a number of mitotic structures (MT ends (during prometaphase), centrosomes, centromeres, kinetochores and spindle MTs). Both the timing of centrosome separation and intrinsic MT assembly rates have been shown to adversely affect mitotic error correction (Ertych et al., 2014; Silkworth et al., 2012). Accordingly, we tested whether centrosome separation (Fig. S1) or MT assembly (Fig. S2) rates were affected by MCAK/Kif2C loss and found no effect. This led us to assume that the effect on congression and mitotic errors was primarily a function of centromere-associated MCAK/Kif2C.

We tested this by anchoring either active (M) or inactive mutant (M^mut^) MCAK/Kif2C motor domain to centromeres using the centromere-binding domain of CENP-B. This is only one of a number of regions where MCAK/Kif2C can localize within the centromere-kinetochore domain but we chose it because we have found that anchoring MT binding proteins (even inactive protein) further distal toward the kinetochore, such as on the Ndc80 protein, tends to produce noticeable defects in control cells.

The GFP-FKBP-MCAK/KIF2C parent cell line was transiently transfected with either Cherry-CPB-M^mut^ (serving as the control) or Cherry-CPB-M (active MCAK/Kif2C activity). These constructs consist of 186–583 of the MCAKKif2C motor domain incorporating AAAAA mutations to prevent inactivation by Aurora B (Andrews et al., 2004). Cherry-CPB-M^mut^ (pMX1400) incorporates the additional mutations H530A/R534A/K537A which effectively inactivate the kinesin motor domain (Woehlke et al., 1997).

**Figure 2.**
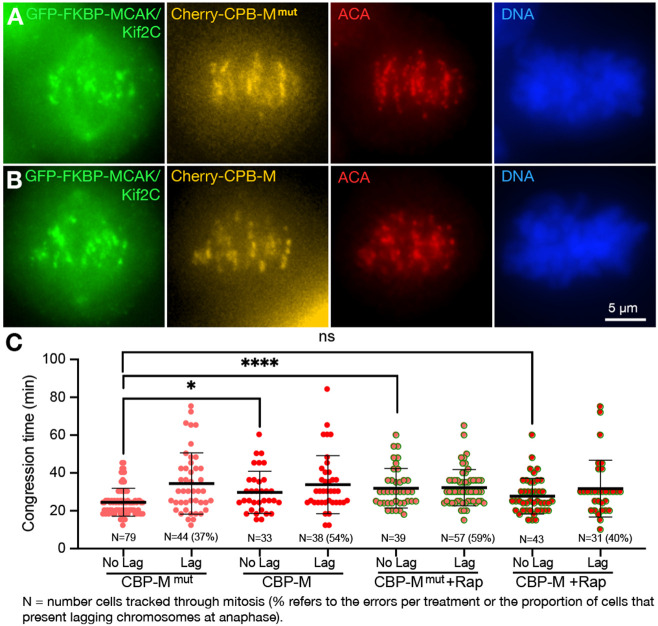
Anchoring the motor domain of MCAK/Kif2C to the centromere can rescue the congression and segregation defects associated with the loss of MCAK/Kif2C in rapamycin. **A.** GFP-FKBP-MCAK/Kif2C parent line transfected with Cherry-CBP-M^mut^ (orange). Endogenous GFP-FKBP-MCAK/Kif2C (green). Immunofluourescently labeled centromeres (red) and DNA (blue) are shown. **B.** GFP-FKBP-MCAK/Kif2C parent line transfected with Cherry-CBP-M (orange). M^mut^ = Hypir mutated inactive motor (control), M = Active MCAK/Kif2C motor. C. Live cells fluorescently tracked through mitosis. Congression times are plotted. Cells that exhibited lagging chromosomes are noted (Lag) and the percentage of tracked cells with lagging chromosomes are indicated (parentheses).

When desired, cells transfected with CPB constructs can also be treated with rapamycin which will relocalize endogenous GFP-FKBP-MCAK/Kif2C to the plasma membrane while having no effect on the MCAK/Kif2C motor domain (M or M^mut^) anchored to the centromere. Representative fixed cells counterstained with Hoechst 33342 (Fig. 2A,B; **blue**) and anti-centromere antibodies (Fig. 2A,B; **red**) cells are shown. Live cells tracked through mitosis are shown in Fig. 2C. The time between NEB and the establishment of the metaphase plate is plotted on the Y-axis. Cells from each treatment were also parsed for the appearance of lagging chromosomes at anaphase. Notably, adding active MCAK/Kif2C activity to centromeres in the presence of endogenous GFP-FKBP-MCAK/Kif2C delayed congression and promoted chromosome segregation errors similar to what we see with OE of full-length MCAK/Kif2C (Fig. 2C, **CBP-M^mut^, salmon circles, vs CBP-M, red circles, No Lag**). However, this condition is rescued if endogenous GFP-FKBP-MCAK/Kif2C is relocalized to the plasma membrane (Fig. 2C, **CBP-M^mut^, salmon circles, vs CBP-M, No Lag, +Rap, red circles, green periphery**). A similar rescue is not seen when CBP-M^mut^ , (a version of the MCAK/Kif2C motor domain lacking MT depolymerizing activity) is treated with rapamycin. Instead the effect is identical to depleting MCAK/Kif2C in the parent cell line because the CPB-M^mut^ construct does not contribute MCAK/Kif2C activity to the centromere. Thus, centromere-localized MCAK/Kif2C is responsible for facilitating congression and this function is disrupted either by loss or excess centromere-associated MCAK/Kif2C activity above endogenous levels.

MCAK/Kif2C has been identified as important for congression by other researchers and we have confirmed and extended these data (Illingworth et al., 2010; Kline-Smith et al., 2004; Ritter et al., 2015). However, the deleterious effect of excess centromere-associated MCAK/Kif2C has only been previously described by our laboratory (Wagenbach et al., 2020). We are puzzled to find that BOTH loss and excess MCAK/Kif2C activity associated with the centromere delays congression and increases the percentage of lagging chromosomes. Because MCAK/Kif2C was thought to be associated with MT plus ends in interphase, we tested centromere separation but found. no difference (Fig. S1A). Subsequently our CRISPR cells revealed that plus tip localization was an artifact of EB3 co-expression (Wagenbach et al., 2023). We have also tested metaphase-to-anaphase timing (Fig. S1B and have found this parameter to be unaffected by changes loss of centromeric MCAK/Kif2C. We did not further pursue centrosome separation or metaphase timing. Instead, we focused on determining the mechanism by which these two conditions (loss and excess of centromeric MCAK/Kif2C) promote chromosome errors and delay congression. To parse the deleterious characteristics of loss and OE of MCAK/Kif2C we looked more closely at centromere motility under these two conditions.

To begin with, we tested whether problems with early attachment were contributing to congression delay. We tracked cells recovering from Monastrol arrest. In such cells, all chromosomes begin the process of congression with attachments at or close to MT plus ends and shorter astral MTs in MCAK/Kif2C OE cells but we have not confirmed this hypothesis.

**Figure 3.**
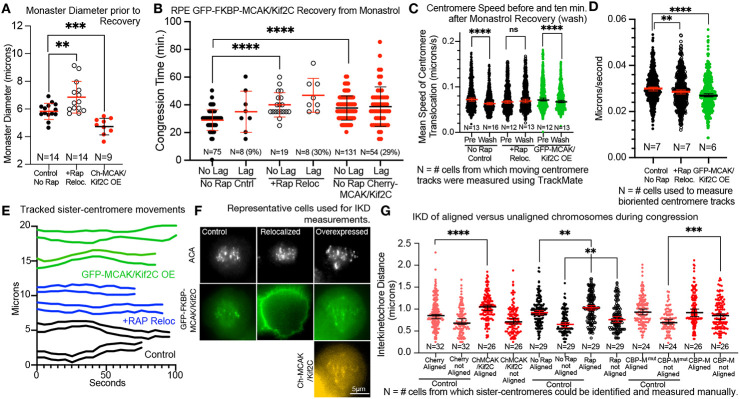
Both loss and excess MCAK/Kif2C increases interkinetochore distance (IKD). **A.** Centromeres settle at different diameters in monastrol-treated cells with MCAK/Kif2C loss leading to a greater diameter (open circles) and additional MCAK/Kif2C leading to reduced diameter (red circles). **B.** Cells experiencing both loss (open circles) and additional MCAK/Kif2C (red circles) exhibit delayed congression and increased segregation errors during recovery from monastrol similar to natural congression. **C.** During early stages of monastrol recovery, kinetochores without MCAK/Kif2C (open circles) appear delayed in forming end-on attachments relative to those with endogenous MCAK/Kif2C (black circles) or excess (green circles). Reduced translocation speed is used as a readout for the establishment of end-on attachments. **D.** Bioriented centromeres translocating on fully bioriented spindles settle into a slow, oscillatory rate of movement. TrackMate tracking speeds are shown. Control centromeres (black circles) translocate slightly more rapidly than either those in +Rap-treated cells (open circles), or those overexpressing GFP-MCAK/Kif2C(green circles); **E.** Representative live bioriented sister centromere pairs from cells with GFP-FKBP-MCAK/Kif2C co-engineered with cherry-CENP-A (control, black traces; relocalized (+rapamycin), blue traces) and the same cells transfected with GFP-MCAK/Kif2C expressing plasmid and co-engineered with cherry-CENP-A (green traces). **F.** Representative fixed cells labeled with ACA in far red (top) and expressing endogenous (left, green), relocalized (middle, green) and endogenous GFP-FKBP-MCAK/Kif2C (right, green) plus excess cherry-MCAK/Kif2C (right, orange). Such cells were used for IKD measurements. **G.** Fixed cells with excess MCAK/Kif2C (red circles, left) have higher IDK in aligned chromosomes relative to cells expressing cherry protein (salmon circles, far left). Cells with no MCAK/Kif2C on centromeres (open circles) because it is relocalized in rapamycin, also exhibit higher IKD on aligned chromosomes relative to control cells (black circles). Cells with active MCAK/Kif2C anchored to the centromere (red circles, right) exhibit a higher IKD relative to control cells (salmon circles, right) but with the marked exception that this is manifest only on chromosomes prior to alignment.

When Monastrol is washed away the results conform to what we see during natural congression: both relocalization and OE of MCAK/Kif2C delay congression, even in cells in which no errors are visible (Fig. 3B, **No Lag**). Both treatments also increase the proportion of lagging chromosomes at anaphase. However, we do note one subtle but likely important difference in centromere motility during recovery from monastrol (Fig. 3C). When centromeres are tracked for speed of movement they move quite rapidly while in (albeit mostly either syntelic or monotelic). Interestingly, cells in the three treatments we tested (Control, Relocalized, MCAK/Kif2C OE) exhibited comparatively different centromere resting positions diameters in monastrol indicating that the three treatments can influence polar ejection forces (using kinetochore position as a readout) although we do not know precisely how (Fig 3A). We hypothesize that this effect may result from longer astral MTs (unattached to kinetochores) in the MCAK/Kif2C relocalized cells monastrol but not for a very long distance. These movements resemble brownian movements. Presumably this motion is characteristic of centromeres that are not bioriented as that would be the one class of centromeres poorly represented in monasters. When monastrol is removed by exchanging with fresh monastrol-free media the centromeres begin exhibit to exhibit slower speeds as they establish bioriented attachments. Establishing bioriented sister-centromeres would involve the establishment of end-on attachments. This activity has been attributed to MCAK/Kif2C by the Draviam lab (Shrestha & Draviam, 2013) which concluded that MCAK/Kif2C activity is required to transition laterally attached chromosomes to end-on attachments. Thus, it is interesting to see that this process appears delayed in cells with MCAK/Kif2C relocalized but not in cells overexpressing MCAK/Kif2C. Eventually, all three treatments lead to slowly oscillating bioriented centromeres (Fig. 3D).

Accumulation of end-on attachments is predicted by a decrease in the speed of movement in both control and MCAK/Kif2C OE cells (Fig. 3C, **black and green circles**) within 10 min. after reversal from Monastrol. In contrast, cells in which MCAK/Kif2C has been relocalized have not transitioned to slower movements within this time period (Fig. 3C, **open circles**) suggesting that they have not significantly transitioned to end-on attachments. Thus, MCAK/Kif2C's primary use of its MT depolymerizing activity is most likely to increase the speed and efficiency of kinetochore attachment to kinetochore MT plus-ends. This observation constitutes one of the very few significant differences observable between MCAK/Kif2C relocalization and overexpression.

Our data supports the conclusion that bioriented end-on attachments are delayed in the absence of MCAK/Kif2C using a live motion readout rather than static images. Chromosome congression can occur with or without end-on attachments (Cai et al., 2009). Furthermore, end-on attachments can be promoted by high polar ejection forces (Drpic et al., 2015), which are evident in the absence of MCAK/Kif2C (Fig 3A, **open circles**). These activities may contribute to the establishment of bi-oriented centromeres in the absence of MCAK/Kif2C.

We revisited the speed of centromere movement in bipolar prometaphase spindles in which all centromeres appear to have established bipolar attachment (Fig. 3D). At this time centromere translocation becomes even slower. However, the mean speed of moving centromeres is modestly faster in control cells (black circles) compared to either depleted (open circles) or MCAK/Kif2C OE cells (green circles). When individual sister centromeres were tracked (Fig. 3E), control centromeres appeared more coordinated (fewer IKD fluctuations) and also more oscillatory (exhibiting more of a sine wave). Accordingly, we investigated the interkinetochore distance (IKD) between sister-centromeres in aligned chromosomes and also in chromosomes that have not yet reached the metaphase plate in naturally congressing chromosomes on bipolar spindles (Fig. 3F and G). Representative individual cells from control (No Rap), relocalized (+Rap) or cells transfected with cherry-MCAK/Kif2C (in addition to endogenous GFP-FKBP-MCAK/Kif2C) are shown in Fig. 3F. Not surprisingly, the IKD is smaller in sister-centromeres prior to alignment. Notably however, both MCAK/Kif2C relocalization and OE resulted in greater IKDs in aligned chromosomes. We have seen this before using MCAK/Kif2C dominant negative constructs (Wordeman et al., 2007). An increase in IKD in the presence of excess MCAK/Kif2C is surprising to us because we saw decreased IKD when we anchored active MCAK/Kif2C to centromeres in CHO cells (Wordeman et al., 2007). Importantly, however, there are two differences between the present study and our previous one. The centromeres in the earlier paper were measured during metaphase which is a time during which anchored MCAK/Kif2C motor domain does not appear to increase IKD. The previous study was performed using CHO cells rather than human cell lines. Our recent findings in human cells, however, are consistent with (Ritter et al., 2015) which showed that a phospho-mutant version of MCAK/Kif2C exhibiting excess activity also increases IKD when introduced into cells. In summary, excess full-length MCAK/Kif2C appears to promote increased IKD primarily in aligned chromosomes and excess MCAK/Kif2C activity anchored at centromeres increases IKD primarily at unaligned centromeres.

**Figure 4.**
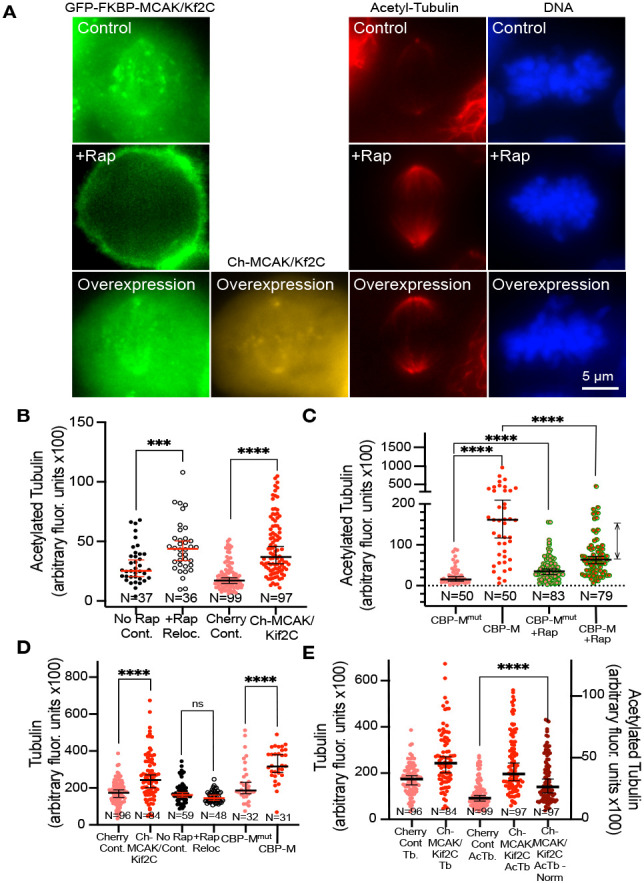
Both excess MCAK/Kif2C activity and loss of MCAK/Kif2C activity raises the level of acetylated tubulin in the spindle. **A.** Representative fixed cells labeled with acetyl-tubulin in the far-red channel (second from right, red). GFP-FKBP-MCAK/Kif2C is shown (left), overexpressed cherry-MCAK/Kif2C (second from left, orange) and DNA (right, blue) are shown. **B.** Loss (open circles) or excess (red circles) MCAK/Kif2C leads to higher levels of acetyl-tubulin in the spindle. **C.** Anchoring excess MCAK/Kif2C activity to centromeres (red circles) significantly raises the level of acetyl-tubulin in the spindle. This can be partially rescued by removing endogenous MCAK/Kif2C from centromeres (red with green perimeter circles). **D.** Treatments that require overnight expression (Ch-MCAK, red, left and CBP-M, red, right) also increase the level of tubulin in the spindle. Treatment with rapamycin (open circles) does not increase tubulin in the spindle. **E.** Normalization of acetyl-tubulin expression relative to the increase in tubulin expression (red circles, left) does not eliminate the significance of the increase in acetyl-tubulin (red circles, right) caused by overnight MCAK/Kif2C expression (dark red circles, right).

It is quite possible that uncoordinated sister-centromere movements (Fig. 3E) seen in both experimental conditions promote increased IDK (Fig 3G) as the sister-centromeres fight for directional dominance. This might exert a pulling force on kinetochore MTs that would stabilize them and impair MT turnover. It has been previously shown that pulling forces on MTs bound to purified kinetochores can stabilize them against disassembly (Akiyoshi et al., 2010). In order to investigate this possibility in cells we measured the fluorescence of acetylated tubulin within the spindle which is known to be primarily associated with kinetochore fibers which are capped (Rasamizafy et al., 2021). We found that MCAK/Kif2C loss, excess MCAK/Kif2C and centromere-associated active MCAK/Kif2C motor domain were each correlated with increased acetylated tubulin in the spindle (Fig. 4).

Figure 4A shows representative fixed cells measured in this assay. Endogenous GFP-FKBP-MCAK/Kif2C is shown (green), along with excess cherry-MCAK/Kif2C (orange) where used. Cells were labeled in far-red with anti-acetyl-tubulin (red) and DNA (Hoechst 33342, blue). Integrated density of fluorescence was measured in the acetyl-tubulin channel using a circle of consistent size (for all cells measured) that is two pixels (0.2μm) from the polar end of the spindle and background subtracted from cytoplasmic non-spindle fluorescence. We observed that cells in which MCAK/Kif2C was removed (Fig. 4B, **open circles**) or overexpressed (Fig. 4B, **red circles, right**) anchored to centromeres (Fig 4C, **red circles, left**) exhibited higher acetylated tubulin fluorescence. Moreover, removal of endogenous GFP-FKBP-MCAK/Kif2C with rapamycin partially rescued (decreased) the acetyl-tubulin fluorescence levels in the spindle (Fig 4C, **red circles with green periphery, right**) similar to the rescue of congression time and errors seen in this regime (Fig. 2C).

Two of our treatments must unavoidably take place over 16 hours (cherry-MCAK/Kif2C expression and cherry-CPB-M expression). We have found previously that alterations in levels of MCAK/Kif2C can promote tubulin expression (Wordeman et al., 2016) in a manner consistent with tubulin autoregulation (Gasic et al., 2019). Thus, we were concerned that increased acetyl-tubulin was a function of increased total tubulin in the spindle. We found that expression of cherry-MCAK/Kif2C and cherry-CPB-M constructs did lead to measurable increases in tubulin fluorescence in the spindle relative to controls (Fig. 4D). This increase did not occur during a four-hour treatment with rapamycin to relocalize GFP-FKBP-MCAK/Kif2C (Fig. 4D, **open circles**). Despite this observation, we do not believe that the increase in acetyl-tubulin in samples subjected to overnight expression can be wholly explained by a simple increase in total tubulin. We have normalized the level of acetyl-tubulin fluorescence to reflect the increase in the total proportion of tubulin and found that spindles over-expressing cherry-MCAK/Kif2C still showed a strong statistically significant increase in acetyl-tubulin (Fig. 4E, **dark red circles**).

**Figure 5.**
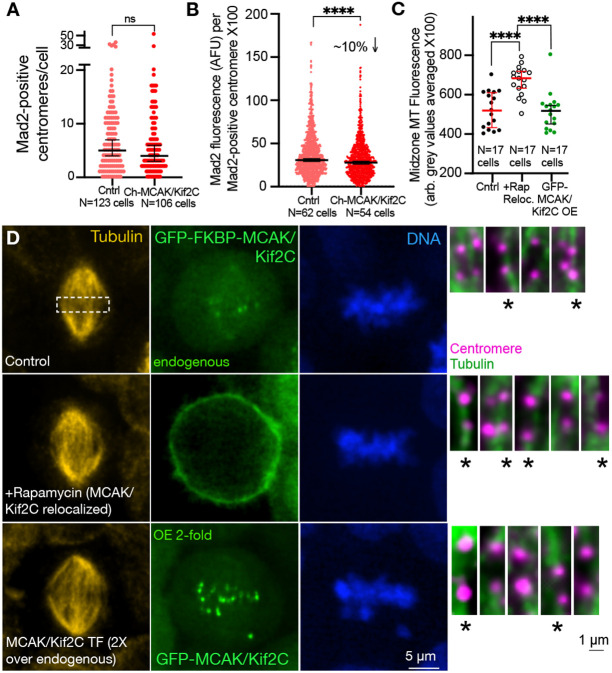
MCAK/Kif2C OE does not significantly increase MAD2 association with centromeres or increase midzone tubulin fluorescence in the vicinity of centromeres. **A.** Number of Mad-2-positive centromeres per prometaphase cell. **B.** Mad2 fluorescence per Mad-2-positive centromere. **C.** Midzone tubulin fluorescence is higher in summed Z-stacks of spindles with relocalized MCAK/Kif2C (open circles) relative to controls (black circles) or GFP-MCAK/Kif2C overexpressing cells (green circles). **D.** Representative summed Z-stacks of mitotic spindles used in the measurements in C. Fluorescence was measured as arbitrary averaged gray values within a consistently sized box (shown top, dotted line box) encompassing the midzone where most of the centromeres were located. Sister-centromeres from the same spindles are shown to the right. Those that possess tubulin fluorescence that invades the region between the sister-centromeres are marked with asterisks (individual sisters were not quantified in this way because they were located in different planes, these are representative examples of fluorescence that might contribute to the levels of tubulin we measure in the summed boxed region). Note that the BFP-labeled cell membrane is generally not visible due to the very high bleach rate of BFP.

Given that changes in IKD can appear to alter spindle stability, we investigated the possibility that loss or excess MCAK/Kif2C influences checkpoint surveillance by Mad2. Both loss or excess MCAK/Kif2C increase IKD and might promote excess tension across sister-centromeres (Bunning & Gupta, 2023). However, this is not likely to be changing the timing of anaphase onset because the checkpoint appears to be active and we do see a delay in congression in our treatments. We have previously shown that loss of MCAK/Kif2C via siRNA temporarily increased the number of Mad2 positive centromeres in prometaphase spindles relative to controls (Domnitz et al., 2012). It appears likely that this increase in MAD2 reflects a delay in establishment of end-on attachments (Fig. 3C). Because MCAK/Kif2C OE does not appear to delay the onset of end-on attachments (Fig. 3C), we predict that we might not see this effect in MCAK/Kif2C OE and we generally do not (Fig. 5A and B). We see no difference in the number of Mad2-positive centromeres in cells over-expressing MCAK/Kif2C (Fig. 5A). However, when we measure the fluorescence of the Mad2-positive centromeres we found a significant but very small (~10%) decrease in Mad2 fluorescence on Mad2-positive centromeres in MCAK/Kif2C OE cells (Fig. 5B). It is not surprising to see a decrease in Mad2 given the effect of MCAK/Kif2C OE on IKD but the loss of Mad2 is so modest that it appears unlikely to be responsible for the appearance of uncorrected errors. Differential Mad2 levels represent only the second distinct difference (other than end-on attachment rate) that we see between depleted centromeres and those associated with excess MCAK/Kif2C.

Each of these treatments (loss or increase in activity) promoted increased IKD in either aligned or unaligned chromosomes (Fig. 3D) which, we hypothesize, may be responsible for increased stabilization of MTs in the kinetochore fibers of the spindle. Commensurate with this effect, these treatments also modestly delayed congression and promoted segregation errors as visualized by lagging anaphase chromosomes. Because the effects of MCAK loss are modest (from our lab and others), these data support the idea, previously proposed by the Draviam lab (Shrestha & Draviam, 2013) which concluded, that MCAK/Kif2C's primary function is to increase the efficiency of the establishment of end-on MT attachments rather than detect and detach aberrant MT attachments. Quantitative measurements of end-on versus lateral interactions between centromeres and MTs is challenging in HCT116 cells. We reasoned that centromeres with a propensity toward lateral MT interactions over end-on MT interaction would be inhibited from clearing the interkinetochore region of tubulin fluorescence as sisters align on the metaphase plate. Consistent with this idea we have recorded greater tubulin fluorescence in the region of the spindle between late prometaphase aligned sister centromeres of spindles with MCAK/Kif2C relocalized as compared to spindles with centromere-associated MCAK/Kif2C (Fig. 5C, **open circles and** D, **middle**). Figure 5D (**top**) shows the boxed region from which tubulin fluorescence was measured in summed projections. Centromeres in spindles without MCAK/Kif2C might tend to associate laterally with MTs in the midzone region to facilitate congression, resulting in increased IKD, as has been seen in oocytes (Kouznetsova et al., 2014). Kinetochore fiber stabilization associated with increased IKD might serve to impair Aurora-dependent MT release in those centromeres that have established end on connections (Liu et al., 2009), leading to increased errors. In contrast, spindles with excess MCAK/Kif2C do not show increased midzone tubulin fluorescence instead appearing similar to control cells (Fig. 5C, **green circles**). In this case, the increased IKD of MCAK/Kif2C overexpressing spindles likely results from excess MCAK/Kif2C depolymerizing activity at the centromere of end-on oriented centromeres. While one might think that this would promote MT detachment, the establishment of overly robust end-on attachments may inhibit MT turnover by a related (and unexpected) mechanism, excess tension leading to kinetochore fiber stabilization. Both these unusual conditions might also override MT release under high tension seen by Chen et al. (Chen et al., 2021). Regardless, although arising from distinct mechanisms, increased IKD appears to stabilize K-fiber MTs in both cases, limiting MT turnover, congression and error-correction.

## Discussion

While we believe we have answered the question of why two opposite treatments produce identical downstream phenotypes, there are two mechanistic questions that remain to be solved. First, we hypothesize that increased IKD is responsible for increased stabilization of kinetochore fiber MTs in both conditions of loss and excess MCAK/Kif2C. Excess stabilization of k-fibers has the potential to promote the anaphase segregation errors seen in both of these conditions. We suspect that the increased IKD we see in both treatments have different mechanistic origins but we do not yet have the spatial resolution to investigate this in live cells. For example, we hypothesize that surplus centromere-associated MCAK/Kif2C leads to high IKD due to excess depolymerization of the kinetochore-bound MTs. It has been shown that MT end-bound MCAK/Kif2C can use depolymerization activity to do work and exert force on centromeres (Oguchi et al., 2011). In control cells this activity appears to be modulated as MCAK/Kif2C often partially vacates aligned centromeres. In contrast, when MCAK/Kif2C is completely missing prior to chromosome alignment we hypothesize that there is a poor force balance between laterally associated sister-centromeres promoted by MCAK/Kif2C loss and end-bound MTs resulting in increased uncoordinated movement and increased IKD as sisters "fight" each other. These ideas are best tested in live cells but we do not yet have super-resolution technology to test this hypothesis.

Second, both excess and loss of MCAK/Kif2C delay congression under conditions in which no subsequent defects in chromosome segregation are detected. We suspect that experimentally treated cells exhibiting delayed congression may possess a greater number of initial erroneous attachments than controls. Many such attachments may be successfully resolved but still exert a delay on congression. We do not yet have an assay to detect transient erroneous prometaphase attachments in live cells although we are working on this. Such attachments may also be resolved in metaphase or anaphase but once all chromosomes have congressed we do not see any delay between metaphase and anaphase (Fig. S1B). We hope to find a means to identify and quantify these transient prometaphase events in live cells in future studies.

In a previous study we demonstrated in CHO cells that addition of a live MCAK/Kif2C motor domain to centromeres promoted rather than suppressed MT turnover in spindles and decreased IKD (Wordeman et al., 2007). In the present study, we used a different construct with a longer neck linker, with improved expression properties, and obtained opposite results in human cell lines. Accordingly, we tested both constructs in our human cell lines and obtained consistent results (increased IKD) suggesting that the altered behavior was more likely correlated with cell line specific characteristics. Anchoring MCAK/Kif2C activity to centromeres is only toxic in the context of the total cellular expression level. In fact, we have shown that additional centromeric MCAK/Kif2C can rescue chromosome segregation defects when expressed in cells with depleted MCAK/Kif2C ((Wagenbach et al., 2020) and this study). It is likely that CHO cells tolerate much higher expression levels than our human cell lines due to their natural metabolic flexibility (Omasa et al., 2010). This could be the source of the discrepancy between the two cell lines.

Using live cell readouts of centromere movement, we have seen that MCAK/Kif2C appears to be required for timely establishment of end-on attachments. This observation is consistent with a previous report that quantified the proportion of lateral and end-on attachments in the context of MCAK/Kif2C (Shrestha & Draviam, 2013). Accordingly, the apparent deficiency of error-correcting activity when MCAK/Kif2C is depleted may actually be a side-effect of the loss of its end-on attachment-promoting activity leading to inhibited error correction. Loss or delay in establishing end-on attachments may promote increased IKD width leading to hyperstabilization of kinetochore fibers. Thus, both loss and overexpression of MCAK/Kif2C may result in k-fiber hyperstabilization that impairs error-correction and slows congression. This explains the similar deleterious effects of both OE and loss of centromere-associated MCAK/Kif2C better than a model invoking selective identification and detachment of erroneous attachments.

## Materials and methods

All authors had access to the study data and reviewed and approved the final manuscript.

### Plasmids

CBP-M (pmx393) and CBP-M^mut^ (pmx1400) were prepared from originally published plasmids, pmx240 and pmx241 (Wordeman et al., 2007). Briefly, a self-complementary linker of sequence GATCCAGTACTCCCGGGAGTACTG was inserted into the BamHI site between the CenpB DNA-binding domain and MCAK neck-motor to add eight additional codons, reducing steric interference between the domains in the protein product.

### Cell culture

CRISPR engineered Hct116 and RPE-hTERT cells were cultured at 37°C and 5% CO_2_ in RPMI medium (#11875093; Gibco) with 2 mM L-Glutamine supplemented with 10% defined fetal bovine serum (#SH30070.03; Hyclone). Transfections were performed using a Nucleofector II (Lonza) according to the manufacturer’s protocol. Detailed instructions on the preparation of CRISPR cell lines are provided here (Wagenbach et al., 2023). Cells were plated onto 35-mm glass-bottom dishes (MatTek) for live imaging or 12 mm, 1.5 coverslips for immunofluorescence (#72290-04, Electron Microscopy Sciences).

### Immunofluorescence

For spinning disk confocal imaging, cells were fixed in prewarmed 4% PFA (#15710; Electron Microscopy Sciences) in PBS for 10 min at 37°C. Cells were then incubated overnight at 4°C with anti-Acetyl tubulin (#T7451; Sigma), DM1α (#CP-06; Sigma) or anti-ACA antibodies with (as needed) Antibody binding was visualized by Dylight 568 secondary antibodies (Jackson) at room temperature. Coverslips were then washed three times in PBS and mounted on slides in Vectashield (VectorLabs).

### Live Imaging

Live imaging was performed on a Nikon CREST spinning disk confocal microscope with 37%C heat block stage (Dagan Corp.). Laser power was ≤5%. Alternatively, some live cell experiments were imaged in a Nikon Biostation IM-Q (Nikon). For Monastrol recovery experiments cells were treated with 100μm Monastrol (#M8515; Sigma) for 4-6 hours and, where necessary, 1μm rapamycin (#553210; Sigma) for 4 hours (added simultaneously). Media was washed out three times and replaced with fresh 37oC media with no drugs. Relocalization is not reversible so no drugs are necessary while imaging. Imaging was performed for 2 hours using 5 min. timepoints. For tracking of natural congression and mitotic timing, cells were imaged for 24 hours at 5 min. timepoints.

### Image analysis and statistics

All images were processed using Nikon NIS-Elements (Nikon) or FIJI software (https://fiji.sc/). Sister centromere movements were tracked by hand using MTrack2 (FIJI). Individual centromeres (meaning not sisters) were tracked using TrackMate (FIJI; (Tinevez et al., 2017)).). Congression was tracked by hand, frame-by-frame from nuclear envelope breakdown to anaphase and the presence or absence of lagging chromosomes (labeled with Hoechst 33342 (#H1399; ThermoFisher) or SPY650-DNA (#CY-SC501; Cytoskeleton, Inc.). When lagging chromosomes were present it was not feasible to count them so the data is scored +/− for the presence of lagging chromosomes.

Fluorescence intensity is measured as arbitrary integrated density units. Measurements within the spindle were background subtracted from cytoplasmic fluorescence. For individual centromeres fluorescence intensity was measured as the brightest point on the centromere and also background subtracted for cytoplasm fluorescence. Acetyl-tubulin and tubulin were quantified as the integrated density of a consistently sized circle of 2μm by 2μm located 0.2 μm from the spindle pole in a summed projection of 21 Z-planes. IKDs were measured by hand using Fiji from stacks consisting of 21 Z planes on only those centromeres that could clearly be identified as sister-centromeres.

For all statistics, the data distributions were assumed to be non-normal and subjected to Mann-Whitney t-tests (Prism 10; Graphpad). Graphs are presented with medians and 95% confidence intervals shown.

## Supplementary Material

Supplement 1

## Data Availability

All data are available in the published article.
