# Supplementary material for: MCAK/Kif2C centromeric activity level tunes K-fiber stability
Source: bioRxiv. 2025 Feb 17:2025.02.16.638494. Preprint. [Version 1] doi: 10.1101/2025.02.16.638494 (PMC11870468; doi:10.1101/2025.02.16.638494)
Supplement: Supplement 1 [file NIHPP2025.02.16.638494v1-supplement-1.pdf]

bioRxiv preprint doi: https://doi.org/10.1101/2025.02.16.638494; this version posted February 17, 2025. The copyright holder for this preprint (which was not certified by peer review) is the author/funder, who has granted bioRxiv a license to display the preprint in perpetuity. It is made available under aCC-BY 4.0 International license.

Supplemental Materials

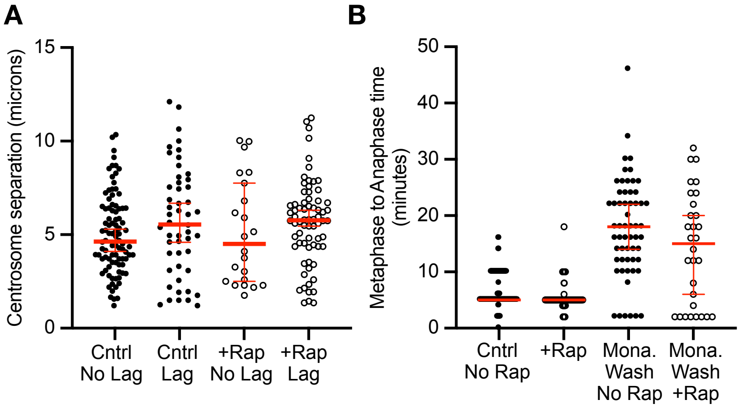

Figure S1. Loss of MCAK/Kif2C had no effect on extent of centrosome separation or metaphase to anaphase timing. **A.** There is no significant difference in centromere separation prior to NEB in cells that manifest lagging chromosomes versus those that do not (black circles). Furthermore, addition of rapamycin to relocalize MCAK/Kif2C does not alter the extent of centrosome separation (open circles). **B.** Timing of the progression from metaphase to anaphase was not significantly different in cells exposed to rapamycin (open circles) to relocalize MCAK/Kif2C as compared to cells with MCAK/Kif2C present (black circles). This proved true for either naturally congressing cells (left) or cells recovering from monastrol treatment (right).

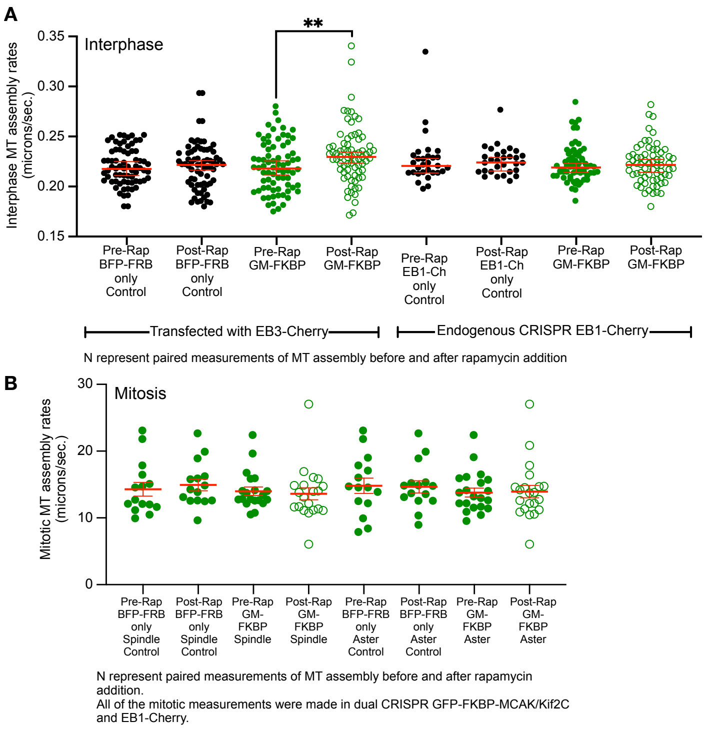

Figure S2. Relocation of MCAK/Kif2C does not affect MT assembly rates unless you measure rates with EB3. **A.** Paired rate measurements of interphase cells transfected with EB3-cherry. Controls (black circles) are cells with no GFP-FKBP-MCAK/Kif2C. Cells with relocalized MCAK/KIf2C (open green circles) do not differ in MT assembly rates from pre-rapamycin (green circles) cells unless EB3-cherry is present. **B.** Paired rate measurements of MT assembly in dual CRISPR GFP-FKBP-MCAK/Kif2C, EB1-cherry cells either within the mitotic spindle (between the centrosomes) or in the asters. Relocalization of MCAK/KIf2C has no effect on MT assembly rates in the spindle or asters (compare pre-treated, green circles, cells with cells that have relocalized in rapamycin, open green circles).
